# Spontaneous Passage of Left Ureteral Multiple Calculi from Caliceal Diverticular Calculi: A Case Report

**DOI:** 10.1089/cren.2018.0081

**Published:** 2019-03-18

**Authors:** Kohei Inui, Masaki Murata, Yuki Nakagawa, Yohei Ikeda, Tsutomu Nishiyama, Yoshihiko Tomita

**Affiliations:** ^1^ Department of Urology, Uonuma Institute of Community Medicine, Niigata University Medical and Dental Hospital, Niigata, Japan.; ^2^ Department of Diagnostic Radiology, Uonuma Institute of Community Medicine, Niigata University Medical and Dental Hospital, Niigata, Japan.; ^3^ Department of Urology, Niigata University Graduate School of Medical and Dental Sciences, Niigata, Japan.

**Keywords:** caliceal diverticular calculi, spontaneous passage, conservative management

## Abstract

Caliceal diverticular calculi are rather fixed and they are rare to be delivered spontaneously. We experienced a case of spontaneous passage of left ureteral multiple calculi from caliceal diverticular calculi. A 65-year-old female suffered from left back pain and was referred to our hospital. She was found to have left ureteral multiple calculi and left caliceal diverticular calculi. After a month observation, all ureteral calculi were delivered spontaneously. Multiple ureteral calculi from caliceal diverticular calculi could be delivered spontaneously and conservative management may be an option for the treatment.

## Introduction

Caliceal diverticula are cystic cavities within renal parenchyma lined by nonsecretory epithelium and communicating with the collecting system through a narrow channel. Patients with symptoms in caliceal diverticular calculi are relatively rare and it is extremely rare to have spontaneous passage. Therefore, surgical managements such as flexible transurethral ureterolithotripsy (f-TUL), percutaneous nephrolithotomy (PCNL), or extracorporeal shockwave lithotripsy (SWL) are usually needed. We report a case of spontaneous passage of left ureteral multiple calculi from caliceal diverticular calculi without surgery.

## A Case Report

**FIG. 1.**
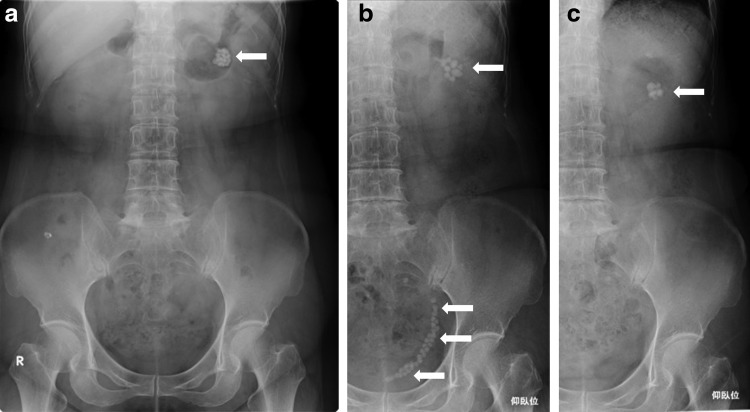
KUB image **(a)** KUB image 8 years before the reference to our hospital reveals many spherical shaped calculi at the upper pole of the left kidney (*arrow*). **(b)** KUB image at the time of visiting our hospital reveals many spherical shaped calculi at the upper pole of the left kidney and beaded calculi that occupied left lower ureter (U2–U3) (*arrows*). The number of the calculi at the upper pole of the left kidney is decreased as compared with that of KUB image 8 years before. **(c)** KUB image 1 month after the first visit showed all ureteral calculi are excreted (*arrow*). KUB, kidney, ureter, and bladder radiograph.

**FIG. 2.**
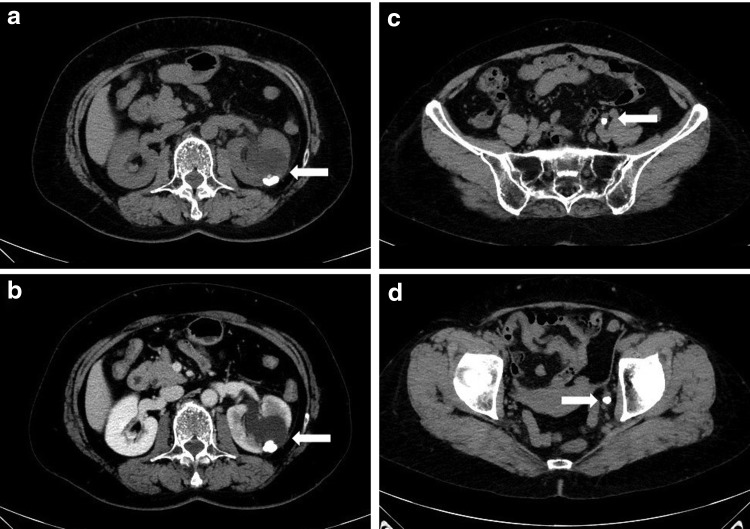
CT image at the time of visiting our hospital. **(a)** Plain CT image revealed a cystic lesion at the upper pole of the left kidney with apparent mural calcification posteriorly (*arrow*). **(b)** Contrast-enhanced CT image revealed that the low attenuation fluid within the cystic cavity when compared with the normally enhancing renal parenchyma confirms the presence of an upper pole caliceal diverticulum (*arrow*). **(c, d)** CT image revealed spherical calculi in the left lower ureter (U2–U3) (*arrow*).

A 65-year-old female was incidentally found to have left renal stones 8 years ago (Fig. 1a). She did not take follow-up for left renal stones. She visited a nearby doctor complaining of left back pain on January 2017. CT, kidney, ureter, and bladder radiograph showed left ureteral multiple calculi and the left caliceal diverticular calculi (Figs. 1b and 2). She was referred to our hospital. She was found to have left ureteral multiple calculi and left renal caliceal diverticular calculi. She was planned for transurethral lithotripsy; however, all ureteral calculi were delivered spontaneously at the time of re-examination 1 month after the first visit (Fig. 1c). Analysis of component of calculus revealed calcium oxalate. She has recovered from left back pain after the passage of all ureteral calculi.

## Discussion

Caliceal diverticulum is a rare disease. It is found by urography 0.33% in children and 0.45% in adults.^1^ There is no gender difference and bilateral difference. It is found in the upper pole calices 48.9% *vs* 29.7% and 21.4% in the central region and lower pole, respectively. Caliceal diverticula are smooth walled nonsecretory cavities within the renal parenchyma and lined with transitional cell epithelium. They receive urine by passive retrograde filling from adjacent collecting system, usually through a narrow infundibulum. Caliceal diverticula are classified into two types. Type I caliceal diverticula are the more common subtype and communicate with a minor calix. Type II caliceal diverticula communicate with a major calix or the renal pelvis itself and are much less common and tend to be larger, are more commonly symptomatic, and are often located in the central part of the kidney.^1^ Urinary stasis may predispose to infection and calculus formation and up to 50% of caliceal diverticula contain calculi or milk of calcium. Caliceal diverticula more commonly contain multiple small calculi rather than a single large calculus; however, the presence of a single calculus within a caliceal diverticulum, although it is extremely rare, can be difficult to differentiate from an obstructed calix.^1^ Although caliceal diverticula are often asymptomatic, they may also be associated with flank pain, urinary tract infection, or hematuria. Asymptomatic patients require no treatment; however, intervention is necessary in symptomatic patients. When the urinary passage to the calix becomes poor because of communicating with the collecting system through a narrow channel, the incidence of calculus formation and urinary tract infection increases with back pain and hematuria.

Active managements such as SWL, f-TUL, PCNL, and laparoscopic extraction are performed as urologic intervention.^2^ When SWL is chosen as a primary intervention, it can provide low stone-free rates ranging from 20% to 58% with symptomatic pain relief in 36%–86% of patients at short-term follow-up.^1^ It is reported that f-TUL is a reasonable minimally invasive treatment option for patients with locations in the upper or central caliceal diverticula and can provide stone-free rates of 84.0% with a symptom-free rate up to 92.0%.^2^ In contrast, the role of laparoscopy is appropriate in the management of stones within anteriorly located or thin layer of parenchyma overlying a large cavity.^3^ Percutaneous management is the most widely used endourologic modality that provides excellent stone-free rates (87.5%–100%).^4^ Percutaneous approach is the generally preferred method for posterior lesions, and anterior diverticula can also be challenging in terms of increased complications such as bleeding from the longer distance that tract has to transverse through the renal parenchyma. Therefore, urologists have to choose a treatment to take into account both the advantages and disadvantages of each treatment. Caliceal diverticular calculi are rather fixed and it is rare to be delivered spontaneously. We experienced a case of spontaneous passage of ureteral multiple calculi from caliceal diverticular calculi. In the present case, all ureteral stones were discharged naturally without treatment. Diverticular calculi in the present case were almost all spherical in shape, so that urine passed the ureters relatively smoothly and the calculi could move through the channel and the ureter. The present case suggests that if caliceal diverticular calculi are spherical, surveillance can be one of the treatment options.

## Abbreviations Used

CT: computed tomography
f-TUL: flexible transurethral ureterolithotripsy
KUB: kidney, ureter, and bladder radiograph
PCNL: percutaneous nephrolithotomy
SWL: extracorporeal shockwave lithotripsy
